# A rapid *in situ *procedure for determination of bacterial susceptibility or resistance to antibiotics that inhibit peptidoglycan biosynthesis

**DOI:** 10.1186/1471-2180-11-191

**Published:** 2011-08-25

**Authors:** Rebeca Santiso, María Tamayo, Jaime Gosálvez, Germán Bou, María del Carmen Fernández, José Luis Fernández

**Affiliations:** 1 INIBIC-Complejo Hospitalario Universitario A Coruña (CHUAC), Unidad de Genética, As Xubias 84, 15006- A Coruña, Spain; 2 Laboratorio de Genética Molecular y Radiobiología, Centro Oncológico de Galicia, C/Dr Camilo Veiras s/n, 15009-A Coruña, Spain; 3 Unidad de Genética, Facultad de Biología, Universidad Autónoma de Madrid, 28049-Madrid, Spain; 4 INIBIC-Complejo Hospitalario Universitario A Coruña (CHUAC), Servicio de Microbiología, As Xubias, 84, 15006-A Coruña, Spain

## Abstract

**Background:**

Antibiotics which inhibit bacterial peptidoglycan biosynthesis are the most widely used in current clinical practice. Nevertheless, resistant strains increase dramatically, with serious economic impact and effects on public health, and are responsible for thousands of deaths each year. Critical clinical situations should benefit from a rapid procedure to evaluate the sensitivity or resistance to antibiotics that act at the cell wall. We have adapted a kit for rapid determination of bacterial DNA fragmentation, to assess cell wall integrity.

**Results:**

Cells incubated with the antibiotic were embedded in an agarose microgel on a slide, incubated in an adapted lysis buffer, stained with a DNA fluorochrome, SYBR Gold and observed under fluorescence microscopy. The lysis affects the cells differentially, depending on the integrity of the wall. If the bacterium is susceptible to the antibiotic, the weakened cell wall is affected by the lysing solution so the nucleoid of DNA contained inside the bacterium is released and spread. Alternatively, if the bacterium is resistant to the antibiotic, it is practically unaffected by the lysis solution and does not liberate the nucleoid, retaining its normal morphological appearance. In an initial approach, the procedure accurately discriminates susceptible, intermediate and resistant strains of *Escherichia coli *to amoxicillin/clavulanic acid. When the bacteria came from an exponentially growing liquid culture, the effect on the cell wall of the β-lactam was evident much earlier that when they came from an agar plate. A dose-response experiment with an *E. coli *strain susceptible to ampicillin demonstrated a weak effect before the MIC dose. The cell wall damage was not homogenous among the different cells, but the level of damage increased as dose increased with a predominant degree of effect for each dose. A microgranular-fibrilar extracellular background was evident in gram-negative susceptible strains after β-lactam treatment. This material was digested by DNase I, hybridised with a specific whole genome probe, and so recognized as DNA fragments released by the bacteria. Finally, 46 clinical strains from eight gram-negative and four gram-positive species were evaluated blind for susceptibility or resistance to one of four different β-lactams and vancomycin, confirming the applicability of the methodology.

**Conclusion:**

The technique to assess cell wall integrity appears to be a rapid and simple procedure to identify resistant and susceptible strains to antibiotics that interfere with peptidoglycan biosynthesis.

## Background

The bacterial cell wall provides shape, with resistance to mechanical stress and to internal osmotic forces. Peptidoglycan or murein is an important component of bacterial cell wall. This forms an enormous network of interlinked chains of alternating subunits of N-acetylglucosamine (NAG) and N-acetylmuramic acid (NAM). Short stem peptides that are attached to NAM are cross-linked to stem peptides from nearby muropeptide strands. Peptidoglycan components are synthesized and assembled in the cytoplasm and transferred to the outer face of the cytoplasmic membrane. There, the penicillin-binding proteins (PBPs) or DD-peptidases catalyze the formation of glycosidic linkages between two muropeptide units producing linear glycan chains and the formation of the peptide bonds between adjacent murein strands, i.e. transpeptidation, resulting in a rigid tridimensional polymer [1-3]. Whereas gram-negative bacteria contain two to five layers of peptidoglycan, gram-positive bacteria exhibit a much thicker cell wall, with teichoic acids attached to the peptidoglycan and to the cytoplasmic membrane. Moreover, there is variability among different species and strains, in the frequency of crosslinking in the peptidoglycan and in the presence of different molecules incorporated into the peptidoglycan [3].

Antibiotics that inhibit bacterial cell wall biosynthesis are the most widely used in current clinical practice [1]. The largest family corresponds to β-lactams, which include penicillins, cephalosporins, carbapenems, monobactams and β-lactamase inhibitors [4]. These antibiotics are analogues of D-alanyl-D-alanine, the terminal aminoacid residues on the precursor NAG/NAM-peptide subunits, thus interacting with the active center of PBPs and covalently reacting with a serine residue. They mainly inhibit the transpeptidation, thus stopping cell growth. Secondarily, a build-up of peptidoglycan precursors triggers murein hydrolases or autolysins, degrading the peptidoglycan and resulting in cell death [5]. In the case of gram-positive bacteria, the teichoic acids that inhibit the autolytic system are lost, so the associated murein hydrolases are activated and degrade the peptidoglycan [3].

Resistance to β-lactams may be due to alterations in the permeability of cell wall, loss or mutation of porins, increased expression of active efflux pumps, and over-synthesis or alteration of PBPs. Nevertheless, the most frequent mechanism is the production of β-lactamases, that hydrolize the β-lactam ring [6,7]. Whereas some β-lactamases degrade specific β-lactams, a great concern exists with respect to extended-spectrum β-lactamases (ESBL) [8].

Besides β-lactams, other antibiotics affect peptidoglycan, acting on different stages of biosynthesis. One of the most relevant is vancomycin, a glycopeptide that binds to terminal D-alanyl-D-alanine from the pentapeptide of the cell wall in gram-positive bacteria, blocking the incorporation of peptides to the cell wall, thus inhibiting peptydoglicane elongation [9]. Vancomycin is the last-line antibiotic for severe gram-positive infections, so the growing increase in resistance is a serious health problem [10]. One mechanism of resistance to vancomycin appears to be alteration to the terminal aminoacid residues of the NAM/NAG-peptide subunits, normally D-alanyl-D-alanine, which vancomycin binds to, decreasing drug affinity [11].

The increase in the number of resistant and multiresistant strains of bacteria is a major concern for health officials worldwide, with severe impact on economy and in public health [12]. Resistance is responsible of thousands of deaths each year. Many of them could be prevented by a rapid detection of the resistant bacteria and prompt administration of the appropriate antibiotic. This is particularly decisive in life-threatening infections or for patients in the intensive care unit [13]. In this case, empirical treatment fails in 20-40% cases, and the change of antibiotic based on late classic antibiogram results may be not successful. Critical clinical situations should benefit from a rapid procedure to evaluate the sensitivity or resistance to antibiotics. Moreover, a correct initial treatment, besides avoiding treatment failure, can prevent the spreading of resistant microorganisms through misuse of antibiotics.

We have recently validated a rapid and simple technique to determine *in situ*, and at the single-cell level, the susceptibility or resistance to quinolones, which induce DNA double-strand breaks [14-16]. The bacteria are immersed in an inert microgel on a microscope slide and incubated in a specific lysis solution that removes the cell wall, membranes and proteins. In quinolone sensitive strains, the DNA is fragmented, showing haloes of peripheral diffusion of DNA fragments emerging from the residual central core, that are visualized under fluorescence microscopy after staining with a sensitive fluorochrome. In case of resistant strains, the nucleoids liberated appear intact, with limited spreading of DNA fibre loops. Our purpose was to adapt this simple technology for a rapid evaluation of the susceptibility or resistance to antibiotics that affect the cell wall.

To evaluate if bacteria are susceptible or resistant to quinolones, it is necessary to find lysing conditions that remove the cell wall from all the cells in the population. If there were cells not lysed or insufficiently lysed, the condition of the DNA that remains inside is unknown. Nevertheless, to assess the efficacy of antibiotics against the cell wall, the lysis must be adapted to only affect those bacteria whose cell wall has been damaged by the antibiotic. The liberation of the nucleoid must be the marker that indicates that the wall has been lysed, i.e., that has been affected by the antibiotic. In case of a resistant strain, bacteria would be practically unaffected by the lysis solution and so do not liberate the nucleoid, which retains its usual morphological appearance under the microscope.

## Results

### Identification of susceptibility-resistance in E. coli strains

**Figure 1:**
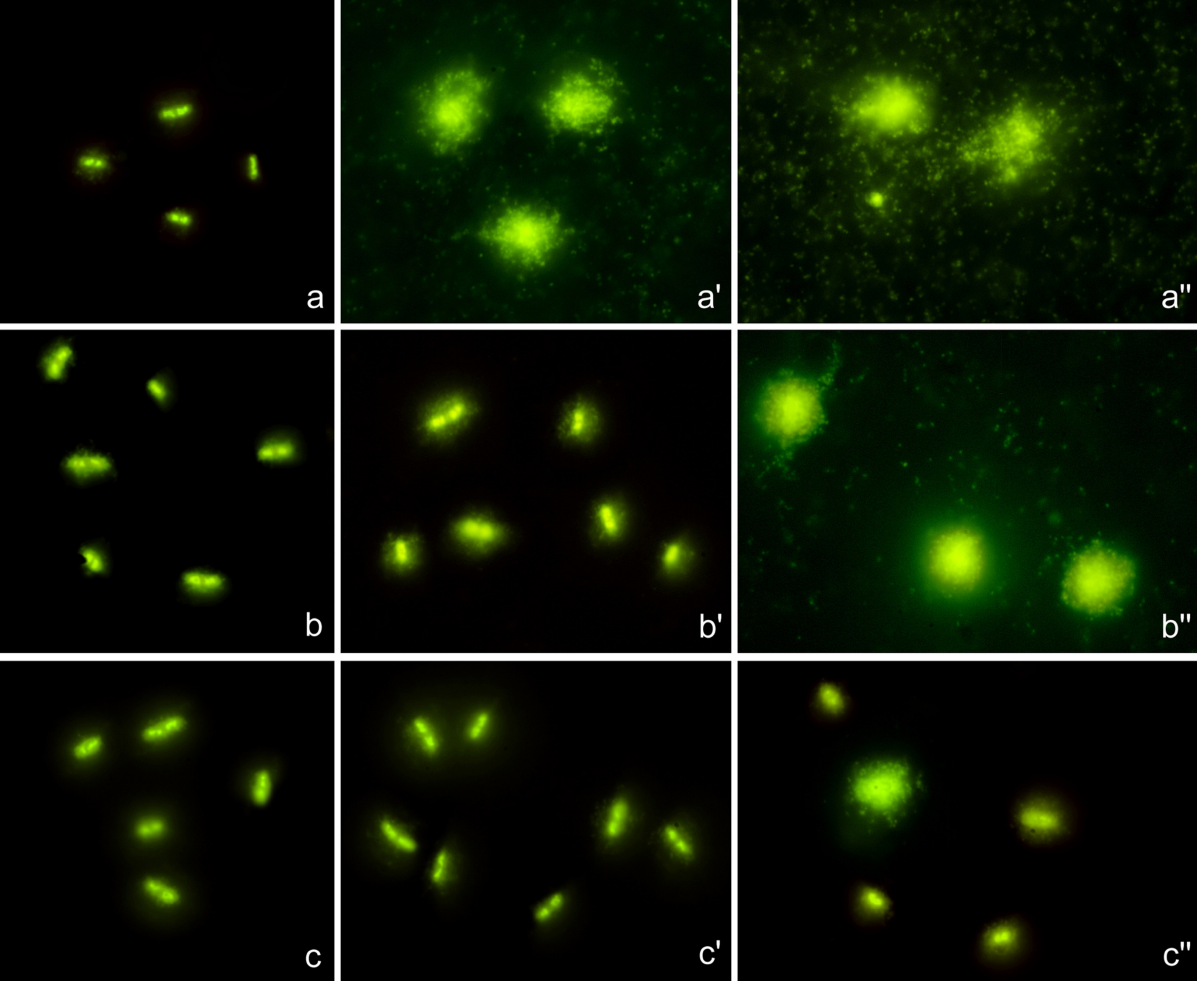
**Images of susceptible (above: a, a', a''), intermediate (medium: b, b', b'') and resistant (below: c, c', c'') strains from *E. coli *incubated with 8/4 μg/ml and 32/16 μg/ml amoxicillin/clavulanic acid and processed by the technique to determine cell wall integrity**. The strain is considered susceptible when its MIC is **≤ **8/4 and resistant when it is **≥ **32/16. **a, b, c**: control, without antibiotic. **a', b', c'**: 8/4 μg/ml; **a'', b'', c''**: 32/16 μg/ml. Controls without antibiotic (**a, b, c**) show the bacteria unaffected by the lysis. After 8/4, only bacteria from the first strain, sensitive, appear lysed, showing the spread nucleoids (**a'**). After 32/16, first and second strains, sensitive and medium, respectively, show to be lysed (**a'' **and **b''**), whereas the third strain, resistant, appears not to be lysed (**c''**). Nevertheless, some isolated bacteria with damaged cell wall are visible. When the antibiotic is effective, besides the liberation of the nucleoids, it is observed a microgranular-fibrilar background of DNA fragments released by the bacteria.

The technique to evaluate cell wall integrity was initially assayed in *E. coli *strains from the clinical microbiology laboratory. Ten strains were processed blind after incubation with amoxicillin/clavulanic acid at doses 0, 8/4 and 32/16 μg/ml, the CLSI breakpoints of susceptibility and resistance, respectively. Example images are presented in Figure 1. Control cultures without antibiotic (Figure 1 a, b, c) showed the bacteria practically unaffected by the lysis. After 8/4 μg/ml, only bacteria from susceptible strains appeared lysed, releasing the nucleoids (Figure 1a'). After 32/16 μg/ml, susceptible and intermediate bacteria appeared to be lysed (Figure 1a'' and 1b''), whereas the resistant strains did not spread their nucleoids (Figure 1c''). Nevertheless, resistance was not homogeneous and some occasional bacteria with damaged cell wall could be visible. Interestingly, a background of extracellular microgranular-fibrilar material released by the bacteria was observed with a density dependent on the efficacy of the antibiotic, thus being especially intense in susceptible strains exposed to relative high doses. The coincidence of the results from the technique and the standard clinical laboratory was absolute, so the two susceptible, the five intermediate and the three resistant strains were correctly identified.

### Nature of the microgranular-fibrilar extracellular background

**Figure 2:**
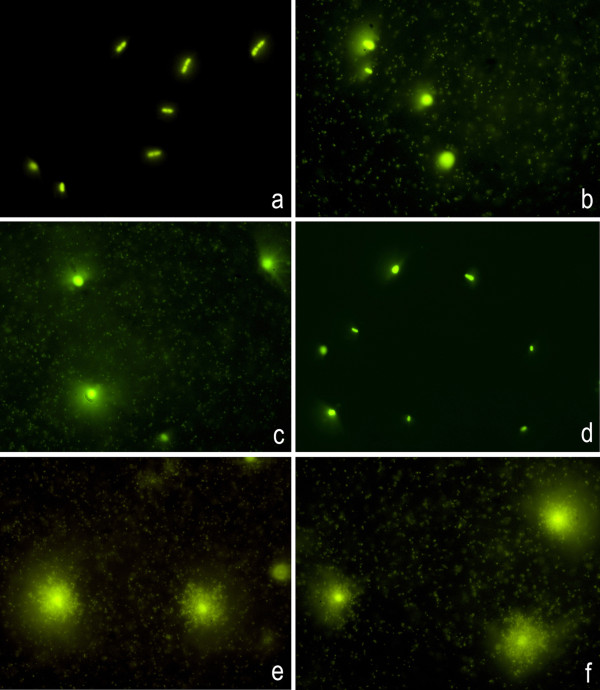
**Nature of the microgranular-fibrilar extracellular background in an *E. coli *strain susceptible to ampicillin, incubated with 32 μg/ml of the antibiotic**. Control culture without ampicillin does not show the microgranular-fibrilar extracellular background (**a**), whereas it is evident in cultures treated with ampicillin (**b**). Incubation of the microgels with specific buffers for DNase I (**c**) or proteinase K (**e**) does not affect the background. The specific proteinase K buffer lyses the bacteria. The background disappears after incubation with DNase I (**d**), but not after proteinase K treatment (**f**).

To investigate the nature of the background, *in situ *digestion with proteinase K and DNase I was performed without a lysis step on microgels prepared from a strain of *E. coli *susceptible to ampicillin and another strain of *A. baumannii *susceptible to imipenem. The microgranular-fibrilar background was evident in the cultures exposed to the antibiotics. This background was not affected by the buffers from the enzymes (Figure 2b, c, e). Treatment with proteinase K was not effective in removing the background (Figure 2f), even when increasing the concentration to 10 mg/ml or diluting in water instead of the buffer, or digesting on the microgel or in cultures fixed in methanol:acetic-acid and spread onto slides. Nevertheless, the background disappeared after incubation with DNase I (Figure 2d), indicating that it corresponded to DNA fragments.

**Figure 3:**
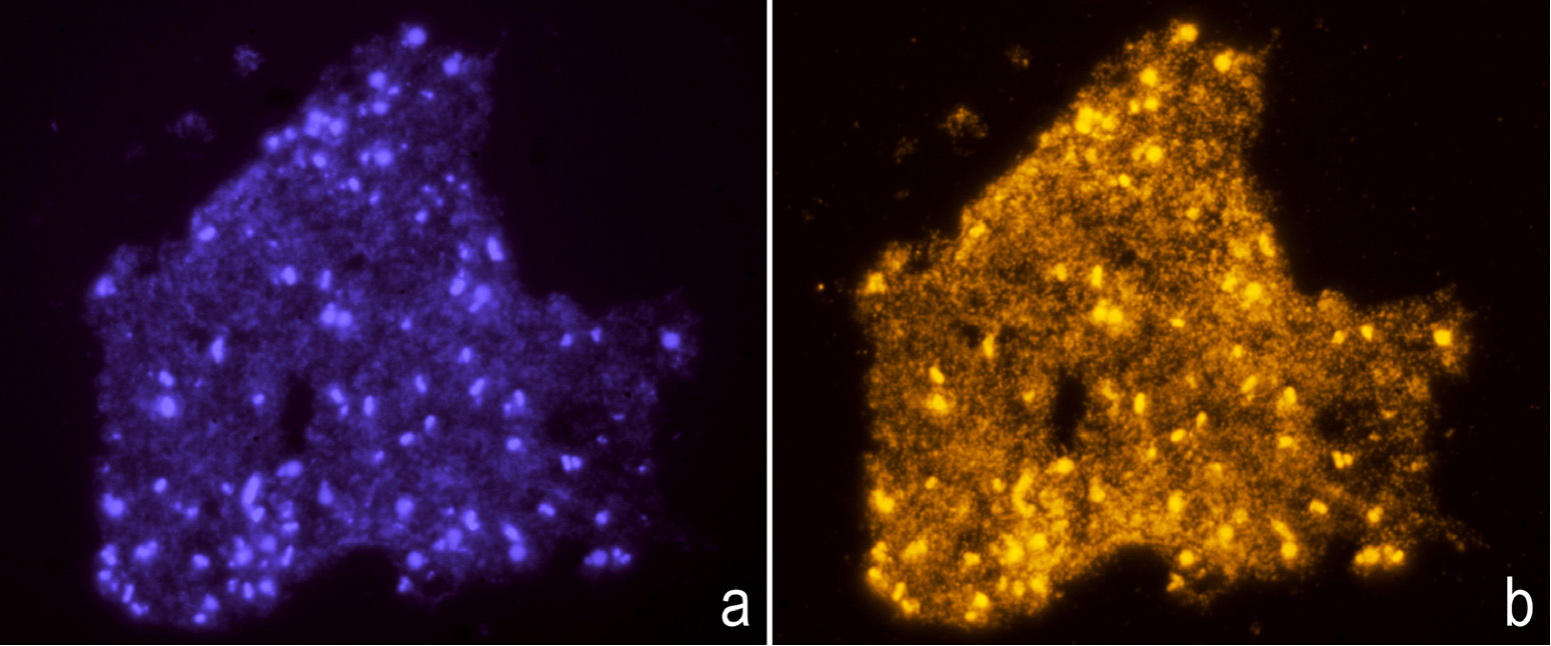
**Fluorescence *In Situ *Hybridization (FISH) with a specific whole genome probe on methanol:acetic acid fixed and spread cultures from *E. coli *treated with ampicillin**. DAPI counterstaining (blue) evidences a faint background of aggregated material that encloses the bacteria that appear more strongly stained (**a**). The whole genome probe, revealed with Cy 3, red, labelled the nucleoids from bacteria and strongly hybridized with the aggregated background (**b**).

To further confirm the previous result, conventional Fluorescence *In Situ *Hybridization (FISH) with a whole genome probe specific to each bacteria, was performed on cultures spread on slides. After fixation in methanol:acetic acid (3:1), the microgranular-fibrilar background tended to aggregate, forming clusters that may enclose the bacteria. DAPI counterstaining penetrated inside the bacteria due to effects on the cell wall, staining the nucleoids. The surrounding background also appeared stained, less intense than the bacteria (Figure 3a). The whole genome probe labelled the nucleoids and hybridized strongly with the aggregated background (Figure 3b), confirming its bacterial DNA nature.

**Figure 4:**
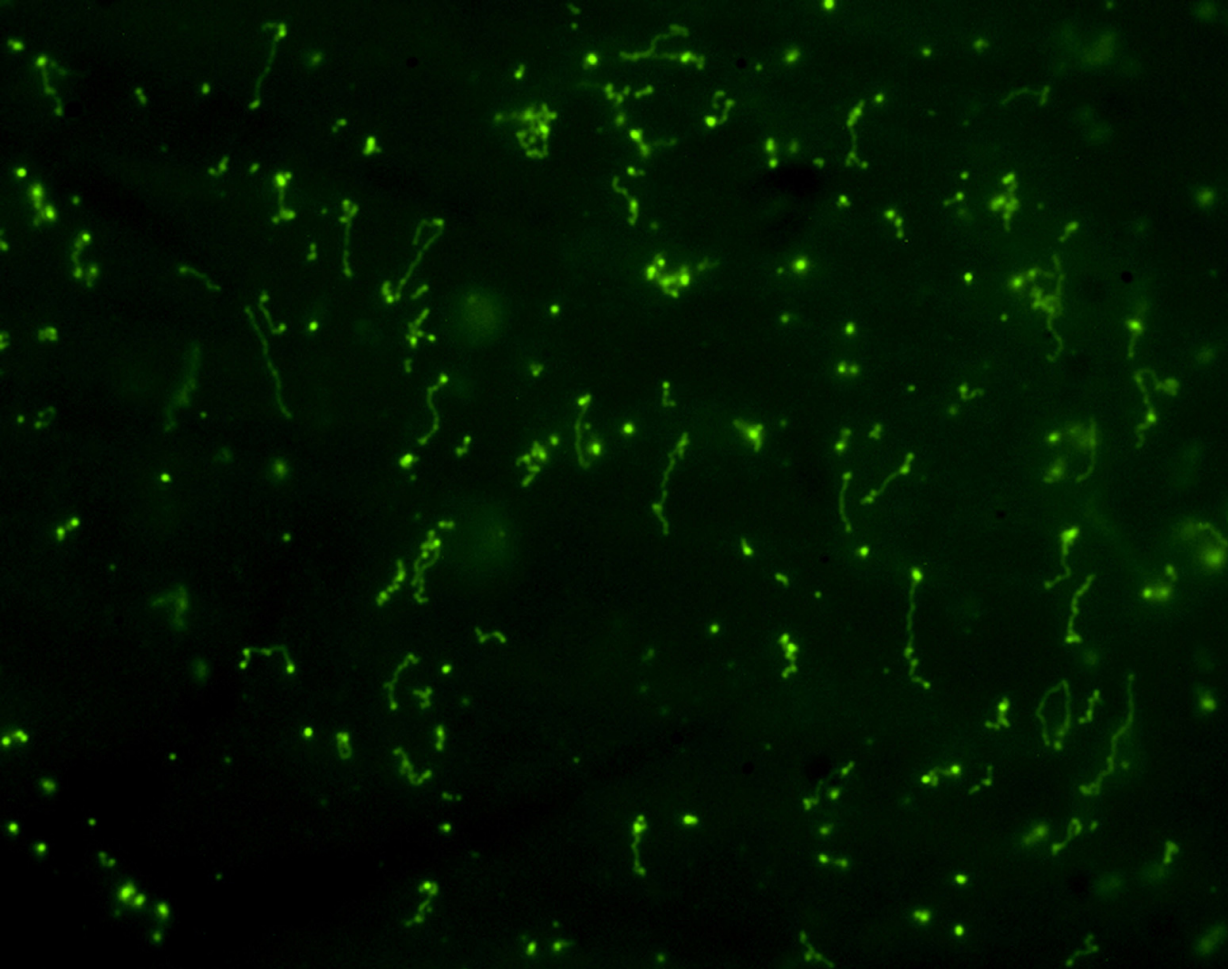
**Background DNA fragments in an *A. baumanii *strain susceptible to imipenem**. The strain was incubated with 0.76 μg/ml of the antibiotic. A high dilution of the culture before being enclosed in agarose microgel allows a more detailed visualization of the extracellular background, after SYBR Gold staining. It is evidenced that the background corresponds to DNA fragments in different levels of spreading, from a punctual appearance to an extended fiber.

In addition, when the culture of *A. baumanni *susceptible to imipenem, was diluted 10 times and immersed in microgel, it allowed us to visualize the background with more detail (Figure 4). The strong staining with the highly sensitive nucleic acid fluorochrome SYBR Gold showed DNA fragments in different levels of spreading, from a dot appearance to an extended fiber.

### Incubation time and culture conditions

To evaluate the influence of the incubation time with the β-lactam, three clinical strains of *E. coli*, one susceptible (MIC: 8/4 μg/ml), one intermediate (MIC: 16/8 μg/ml) and one resistant (MIC: > 64/32 μg/ml), were treated with amoxicillin/clavulanic acid at doses 0, 8/4 and 32/16 μg/ml for 75 min. The origin of the culture before antibiotic treatment, either growing from 24 h in agar dish or exponentially growing in liquid broth was also assessed.

When coming from a culture growing 24 h in agar plate, the susceptible strain after 20 min with the high dose showed an initial and slight cell lysis with faint background of extracellular DNA fragments. With the low dose, the effect was evident after 40 min. After 60 min the effect was the maximum (like Figure 1 a'). The intermediate strain revealed a delayed and slight effect only after the high dose for 60 min, being more evident after 75 min. The resistant strain never showed an effect, although some cells appeared slightly lysed at 75 min after the high dose (like Figure 1c'').

When the bacteria came from exponentially growing liquid culture, the effect on the cell wall was evident much earlier. After 10 min, the susceptible strain showed clear effects, small at 8/4 dose but pronounced with the 32/16 dose. After 30 min, the effect was intense at 8/4 dose, similar to that on the culture coming from agar dish after 60 min incubation. The intermediate strain revealed a weak effect only after 30-40 min with the high dose, being more evident after 60 min. As in the case of cultures coming from agar plate, the resistant strain never showed an effect, although a few cells appeared slightly lysed after 60 min.

### Dose-effect

**Figure 5:**
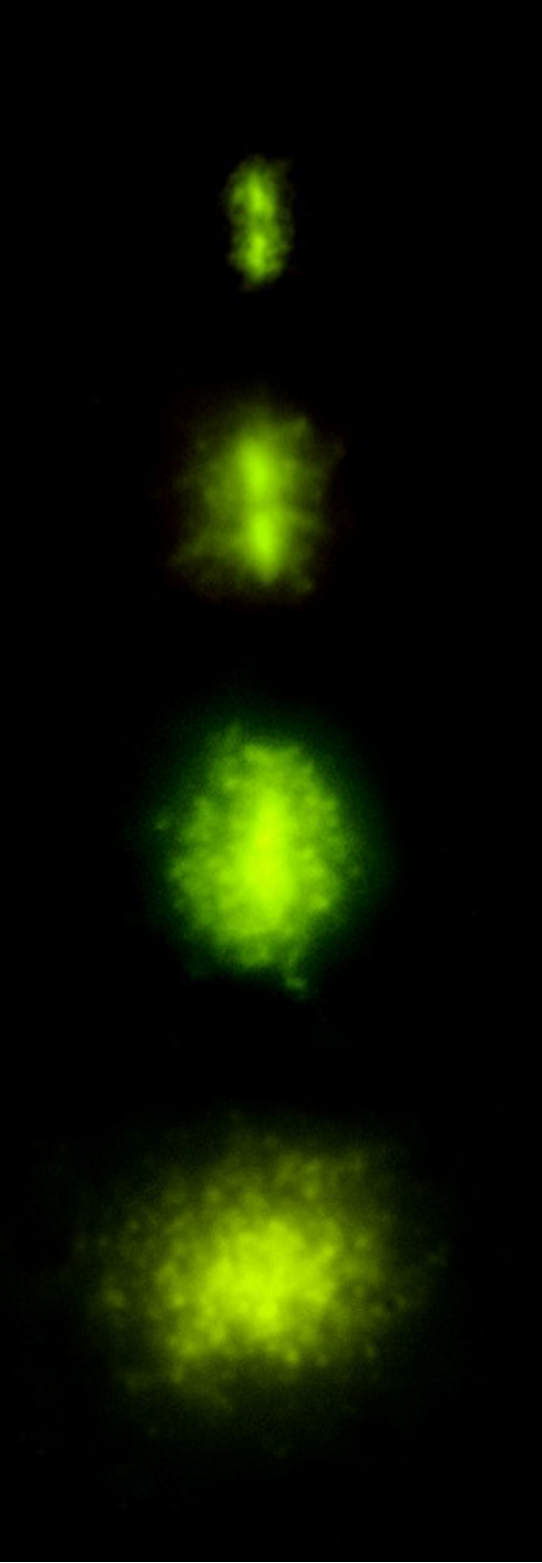
**Categories of *E. coli *exposed to ampicillin, after processing by the procedure to determine cell wall integrity, determined by the spreading of the internal nucleoid**. From above to below: Unaffected, Weakly affected, Strongly affected, Strongly affected without recognizable cell body.

**Figure 6:**
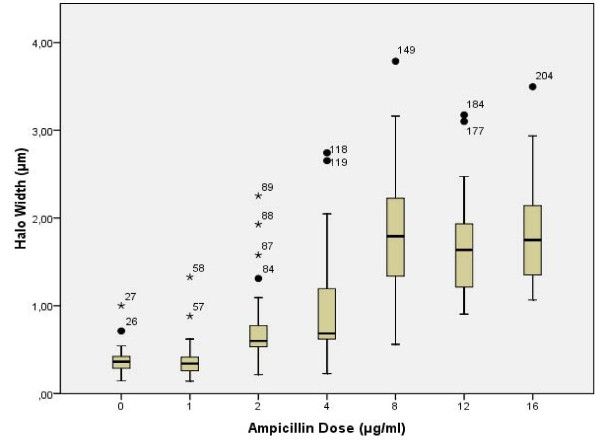
**Halo width of spreading of the nucleoids from the bacterial body from *E. coli *after increasing doses of ampicillin**.

One *E. coli *strain sensitive to ampicillin (MIC: 4 μg/ml) was exposed to increasing doses of the antibiotic to evaluate the effect on the cell wall. Qualitatively, four categories could be easily established (Figure 5). Unaffected bacteria only revealed a background effect of the lysing solution, generally with a very restricted spreading of some DNA fibres from the bacterial body. Affected bacteria could show a weak effect, with a small peripheral halo of DNA loops emerging from the bacterial body, or a strong effect, where the cells are lysed and the nucleoid is completely released from the residual bacterium. The effect may be even stronger, so the residual core from the bacterium is not recognized inside the spread nucleoid. The measure of the halo width of spreading of the nucleoid established 0.40 μm as the limit of halo size between unaffected and small cell wall damage, whereas it was 0.80 μm between low and high cell wall damage. Furthermore, the average halo width of spreading of the nucleoids provided a quantitative estimation of the effect on the cell wall (Figure 6).

**Figure 7:**
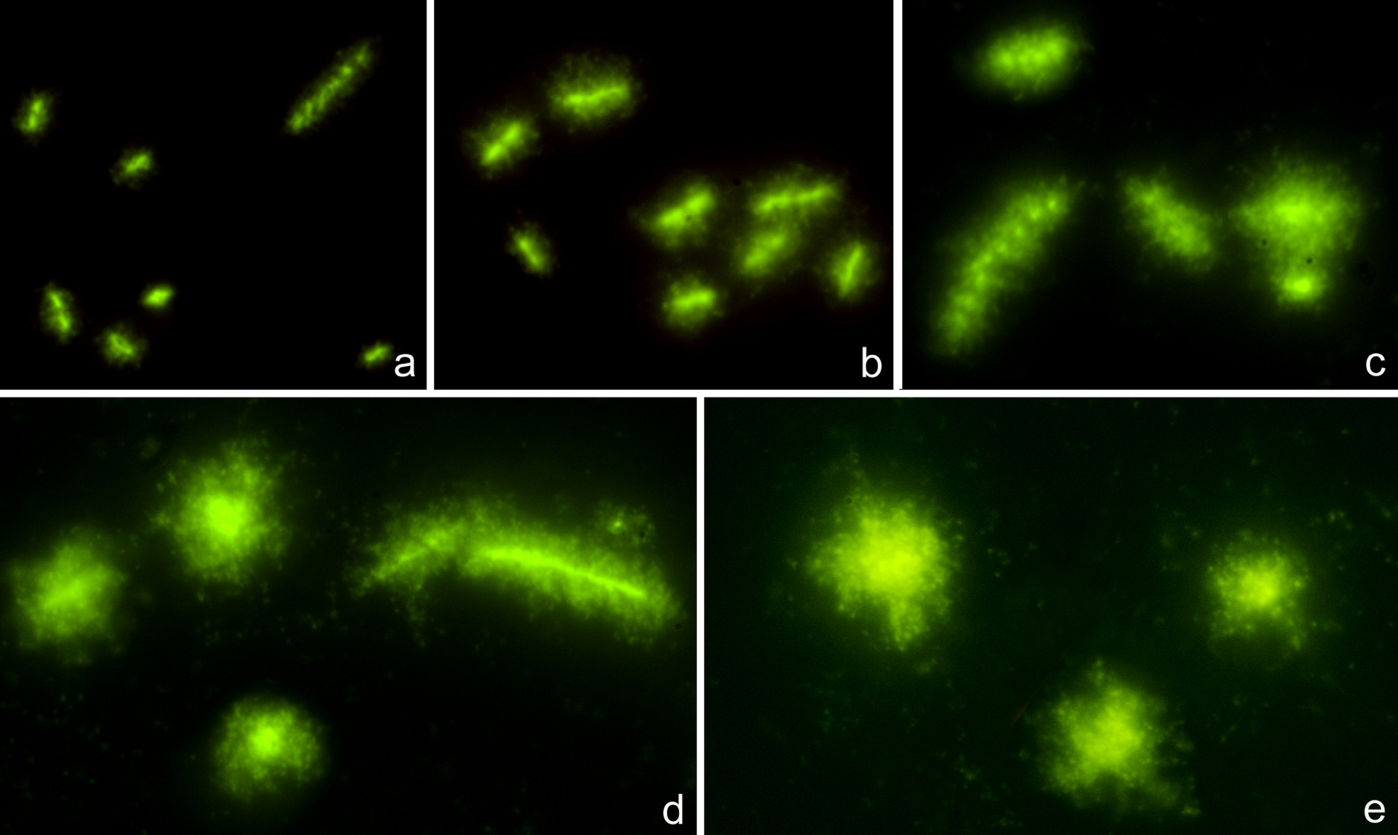
**Representative images of the effect of increasing doses of ampicillin in a susceptible strain of *E. coli***. **a**: control, 0 μg/ml; **b**: 2 μg/ml; **c**: MIC dose, 4 μg/ml; **d**: 8 μg/ml; **e**: 12 μg/ml.

**Figure 8:**
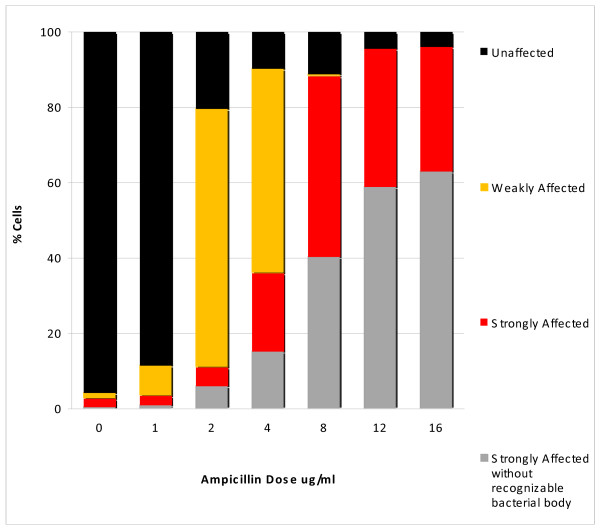
**Proportions of the different categories of cell wall damage after increasing dose of ampicillin in susceptible *E. coli *cultures**.

Figure 7 shows representative images, whereas Figure 8 reveals the proportion of the different categories of cell wall damage with increasing doses of ampicillin. A slight effect was detected in most of bacteria after 2 μg/ml, which should not be enough to prevent viability in most of them when incubated in medium without antibiotic. After the MIC dose, almost all cells showed strong cell wall damage, with a predominance of those where the residual cell core is not visualized within the nucleoid after the highest doses (Figures 7, 8). In fact, despite the similar halo width of the spread nucleoids after 8, 12 and 16 μg/ml (Figure 6), the fraction of cells where the core from the bacterium is not recognized inside the nucleoid increased progressively (Figures 7, 8). The background of DNA fragments was scarce at the MIC dose, increasing with the higher doses.

### Evaluation of clinical strains

**Figure 9:**
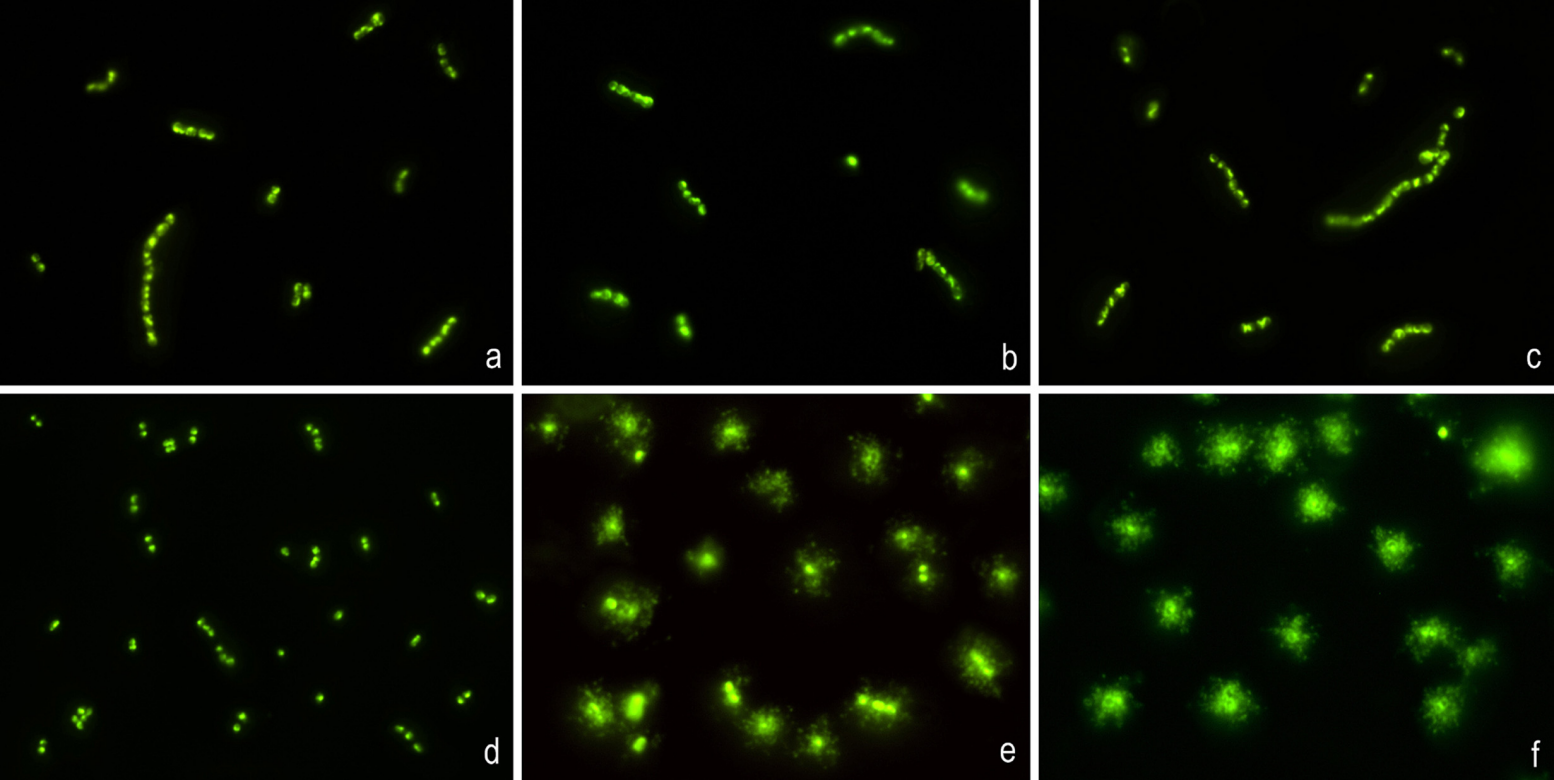
**Two strains of *Enterococcus spp*., one resistant (above: a, b, c) and one susceptible (below: d, e, f), exposed to vancomycin, 0 μg/ml (left: a, d), 4 μg/ml (medium: b, e) and 32 μg/ml (right: c, f) and processed by the procedure to assess cell wall integrity**. Whereas the resistant strain does not show lysis after vancomycin doses, the sensitive strain is clearly affected. There is no evidence of background of extracellular DNA fragments.

To extend the applicability of the methodology, 46 clinical strains from medically relevant species, were evaluated blind for susceptibility or resistance to one of four different β-lactams. Eight gram-negative and four gram-positive species were assayed (Table 1). Vancomycin was also tested in gram positive enterococci and staphylococci, due to its great clinical relevance (Figure 9). The strains were incubated with the CLSI breakpoint concentrations of susceptibility (low dose) and resistance (high dose) of each antibiotic. The strain was considered susceptible if nucleoids were released after the low dose, intermediate if nucleoids were released only after the high dose, and resistant if nucleoids were not released after the high dose. The results obtained with the procedure always coincided with those from the standard techniques from the clinical laboratory. The concentration where the presence of the background of DNA fragments was observed coincided with that where nucleoids were released, in gram-negative strains. Nevertheless, in spite of releasing of nucleoids, the background of DNA fragments was very scarce or undetectable in susceptible gram-positive strains at the doses employed (Table 1 Figure 9).

**Table 1:** Microorganisms evaluated for susceptibility-resistance to antibiotics that inhibit peptidoglycan synthesis

Gram	Bacteria	Antibiotics- CLSI MIC Interpretative Standard (μg/mL)	CLSI Category	MIC (μg/ml)	Drug concentration at which the nucleoids were spread - and DNA fragments were released
Gram -	*Acinetobacter baumannii*	Imipenem: ≤ 4 - 8 - ≥16 (SI, R)	Susceptible	2	4-4

Gram -	*Acinetobacter baumannii*	Imipenem: ≤ 4 - 8 - ≥16 (SI, R)	Intermediate	8	16-16

Gram -	*Acinetobacter baumannii*	Imipenem: ≤ 4 - 8 - ≥16 (SI, R)	Resistant	> 16	No nucleoids-No fragments

Gram -	*Acinetobacter baumannii*	Imipenem: ≤ 4 - 8 - ≥16 (SI, R)	Resistant	> 16	No nucleoids-No fragments

Gram -	*Acinetobacter baumannii*	Ceftazidime: ≤ 8 - 16 - ≥32 (S, I, R)	Susceptible	4	8-8

Gram -	*Acinetobacter baumannii*	Ceftazidime: ≤ 8 - 16 - ≥32 (S, I, R)	Intermediate	12	32-32

Gram -	*Acinetobacter baumannii*	Ceftazidime: ≤ 8 - 16 - ≥32 (S, I, R)	Resistant	> 256	No nucleoids-No fragments

Gram -	*Enterobacter cloacae*	Imipenem: ≤ 1 - 2 - ≥4 (S, I, R)	Susceptible	< 1	1-1

Gram -	*Enterobacter cloacae*	Imipenem: ≤ 1 - 2 - ≥4 (S, I, R)	Susceptible	< 1	1-1

Gram -	*Enterobacter cloacae*	Ceftazidime: ≤ 4 - 8 - ≥16 (S, I, R)	Susceptible	< 1	4-4

Gram -	*Enterobacter cloacae*	Ceftazidime: ≤ 4 - 8 - ≥16 (S, I, R)	Susceptible	< 1	4-4

Gram -	*Escherichia coli*	Ampicillin: ≤ 8 - 16- ≥32 (S, I, R)	Susceptible	2	8-8

Gram -	*Escherichia coli*	Ampicillin: ≤ 8 - 16- ≥32 (S, I, R)	Intermediate	12	16-16

Gram -	*Escherichia coli*	Ampicillin: ≤ 8 - 16- ≥32 (S, I, R)	Resistant	256	No nucleoids-No fragments

Gram -	*Escherichia coli*	Ceftazidime: ≤ 4 -8- ≥16 (S, I, R)	Susceptible	0.25	4-4

Gram -	*Escherichia coli*	Ceftazidime: ≤ 4 -8- ≥16 (S, I, R)	Resistant	32	No nucleoids-No fragments

Gram -	*Klebsiella oxytoca*	Imipenem: ≤ 1 - 2 - ≥4 (S, I, R)	Susceptible	< 1	1-1

Gram -	*Klebsiella oxytoca*	Ceftazidime: ≤ 4 - 8 - ≥16 (S, I, R)	Susceptible	< 1	4-4

Gram -	*Klebsiella spp*.	Imipenem: ≤ 1 - 2 - ≥4 (S, I, R)	Susceptible	< 1	1-1

Gram -	*Klebsiella spp*.	Imipenem: ≤ 1 - 2 - ≥4 (S, I, R)	Susceptible	< 1	1-1

Gram -	*Klebsiella spp*.	Imipenem: ≤ 1 - 2 - ≥4 (S, I, R)	Susceptible	< 1	1-1

Gram -	*Klebsiella spp*.	Ceftazidime: ≤ 4 - 8 - ≥16 (S, I, R)	Intermediate	8	16-16

Gram -	*Klebsiella spp*.	Ceftazidime: ≤ 4 - 8 - ≥16 (S, I, R)	Resistant	> 16	No nucleoids-No fragments

Gram -	*Klebsiella spp*.	Ceftazidime: ≤ 4 - 8 - ≥16 (S, I, R)	Resistant	> 16	No nucleoids-No fragments

Gram -	*Morganella morganii*	Imipenem: ≤ 1 - 2 -≥4 (S, I, R)	Intermediate	2	4-4

Gram -	*Morganella morganii*	Ceftazidime: ≤ 4 - 8 -≥16 (S, I, R)	Susceptible	< 1	4-4

Gram -	*Proteus mirabilis*	Imipenem: ≤ 1 - 2 - ≥4 (S, I, R)	Intermediate	2	4-4

Gram -	*Proteus mirabilis*	Ceftazidime: ≤ 4 - 8 - ≥16 (S, I, R)	Susceptible	< 1	4-4

Gram -	*Salmonella spp*	Imipenem: ≤ 1 - 2 - ≥4 (S, I, R)	Susceptible	< 1	1-1

Gram -	*Salmonella spp*	Ceftazidime: ≤ 4 - 8 - ≥16 (S, I, R)	Susceptible	< 1	4-4

Gram+	*Enterococcus faecalis*	Ampicillin: ≤ 8 - ≥16 (S, R)	Susceptible	4	8-No fragments

Gram+	*Enterococcus faecalis*	Penicillin: ≤ 8 - ≥16 (S, R)	Susceptible	2	8-No fragments

Gram+	*Enterococcus faecalis*	Vancomycin: ≤ 4 -8/16- ≥32 (S, I, R)	Resistant	> 32	No nucleoids-No fragments

Gram+	*Enterococcus faecium*	Ampicillin: ≤ 8 - ≥16 (S, R)	Resistant	> 32	No nucleoids-No fragments

Gram+	*Enterococcus faecium*	Penicillin: ≤ 8 - ≥16 (S, R)	Resistant	> 32	No nucleoids-No fragments

Gram+	*Enterococcus faecium*	Vancomycin: ≤ 4 -8/16- ≥32 (S, I, R)	Susceptible	< 1	4-No fragments

Gram+	*Enterococcus spp*.	Ampicillin: ≤ 8 - ≥16 (S, R)	Susceptible	< 1	8-No fragments

Gram+	*Enterococcus spp*.	Ampicillin: ≤ 8 - ≥16 (S, R)	Intermediate	12	16-No fragments

Gram+	*Enterococcus spp*.	Ampicillin: ≤ 8 - ≥16 (S, R)	Resistant	> 16	No nucleoids-No fragments

Gram+	*Enterococcus spp*.	Penicillin: ≤ 8 - ≥16 (S, R)	Susceptible	2	8-No fragments

Gram+	*Enterococcus spp*.	Penicillin: ≤ 8 - ≥16 (S, R)	Resistant	> 16	No nucleoids-No fragments

Gram+	*Enterococcus spp*.	Vancomycin: ≤ 4 -8/16- ≥32 (S, I, R)	Susceptible	2	4-No fragments

Gram+	*Enterococcus spp*.	Vancomycin: ≤ 4 -8/16- ≥32 (S, I, R)	Resistant	> 32	No nucleoids-No fragments

Gram+	*Enterococcus spp*.	Vancomycin: ≤ 4 -8/16- ≥32 (S, I, R)	Resistant	> 32	No nucleoids-No fragments

Gram+	*Staphylococcus aureus*	Vancomycin: ≤2 - 4-8 - ≥16 (S, I, R)	Susceptible	< 1	2-No fragments

Gram+	*Staphylococcus aureus*	Vancomycin: ≤2 - 4-8 - ≥16 (S, I, R)	Susceptible	< 1	2-No fragments

## Discussion

We have adapted our methodology for assessment of susceptibility or resistance to quinolones [14-16] to develop a simple and rapid procedure to determine susceptibility or resistance to antibiotics that act at the cell wall. This objective may have great application since these are by far the most frequent antibiotics employed all over the world [1].

To assess quinolone activity, the lysing conditions of bacteria were designed to remove the cell wall and membranes from all the cells equally, leaving the cell wall affected or intact [14,15]. In the case of antibiotics that act at the cell wall, the lysis must be adapted to only affect to those bacteria whose cell wall had been damaged by the antibiotic. These bacteria would have a debilitated wall, which would be much more sensitive to the lysing conditions designed to such an effect. The lysis affects the cells differentially, depending on the integrity of the wall. If the bacterium is susceptible, the weak cell wall is affected by the lysing solution so that the nucleoid of DNA contained inside the bacterium is released and spread. On the other hand, if the bacterium is resistant to the antibiotic, it would be virtually unaffected by the lysis solution and does not liberate the nucleoid, remaining essentially with its usual morphological appearance.

The present work describes a logical sequence of experiments to achieve the objective of developing a simple and rapid procedure to determine susceptibility or resistance to antibiotics that act at the cell wall. Firstly, it was necessary to demonstrate the ability of the procedure to discriminate susceptible, intermediate and resistant strains. This was confirmed in clinical *E. coli *strains. As a consequence of the images obtained and to provide an adequate interpretation, the nature of the microgranular-fibrilar extracellular background observed in the preparations was recognized. The influence of culture conditions and incubation time on the observed effect was explored, allowing a detailed dose-effect analysis of the β-lactam, establishing categories of cell wall damage. Finally, the utility of the methodology was demonstrated and extended to clinically relevant gram-negative and gram-positive microorganisms. To our knowledge, there are no references on our work to discuss, given the novelty of the technique.

The procedure was able to distinguish *E. coli *strains that were susceptible, intermediate and resistant to amoxicillin/clavulanic acid. Susceptible strains appeared lysed releasing the nucleoid after the cut-off dose point of susceptibility (8/4 μg/ml), whereas intermediate strains only were affected by the threshold dose of resistance (32/16 μg/ml). Intermediate strains were only lysed after this latter dose. From the clinical point of view, besides the control 0 dose, the assay with the breakpoint dose of susceptibility could be enough to discriminate susceptible from non-susceptible strains. This may make the analysis of lots of strains very accessible with the procedure. In fact, the important fact for the therapeutic decision is the differentiation between susceptible or non-susceptible. Intermediate strains should not be treated with the antibiotic, being preferable to use an alternative one to which they are totally susceptible.

The growing stage of the bacterial population must influence the efficacy of the antibiotic, affecting the kinetics of action. In fact, cells that are not growing or in stationary phase extraordinarily decrease the susceptibility to β-lactams [17]. Moreover, the effect at the cell wall of the antibiotic is a dynamic process, concentration and time-dependent.

From the experiment of incubation time, it is deduced that to discriminate with accuracy the susceptible strains from the rest it is enough, in a practical clinical approach, to assess the control 0 dose and the CLSI cut-off dose for susceptibility, incubating with the antibiotic for 60 min in case of cultures growing 24 h in agar plate, as usual in the standard clinical microbiology laboratory. If the cultures were exponentially growing in liquid medium, the incubation time with the antibiotic may be decreased for 30 min. We have observed that the greater the ageing of the culture in agar plate, or when the culture is achieving the stationary phase of growth, the longer the incubation time necessary to observe the effect of the antibiotic, even several hours. To evaluate clinical strains using the technique to assess the integrity of the cell wall, it is mandatory to simultaneously process a sensitive, an intermediate and a resistant strain as controls of the activity of the antibiotic and the efficacy of the technique.

Sensitive strains from gram-negative bacteria assayed showed a background of extracellular microgranular-fibrilar material, its concentration being dose and time dependent. This material corresponded to DNA fragments released by the bacteria, since it was digested by DNase I and hybridized with a specific whole genome probe, being clearly visualized with high sensitive DNA dyes, i.e. SYBR Gold. It is interesting to note that this background of DNA fragments was practically undetectable in gram-positive strains, despite being susceptible to β-lactams or vancomycin. Moreover, it was also undetected in the same bacteria after quinolone treatment in susceptible strains, as evidenced in our previous works with the procedure [15,16]. This fact suggests that the release of DNA fragments could be specific to cell wall directed antibiotics or β-lactams at least.

This interesting phenomenon requires a deeper study in future works, to address the mechanisms and kinetics of production. DNA fragmentation must be a secondary effect, after cell wall damage. It could be a passive result of attack by DNases or reactive species of oxygen (ROS) liberated in the affected bacteria, or it could be active, a consequence of an apoptotic-like process triggered after cell wall damage. Considering to the first possibility, it has been recently reported that, unlike bacteriostatic antibiotics, β-lactams induce the formation of ROS in gram-negative and gram-positive microorganisms [18]. Hydroxyl radicals should attack proteins and DNA, possibly inducing DNA breakages, resulting in death of the bacteria. This response was also found with other bactericidal antibiotics, like fluoroquinolones. Possibly, the increased permeability of the cell wall that would result after impairment of peptidoglycan biosynthesis by the β-lactams, would allow the release of DNA fragments to the medium. Nevertheless, the DNA fragments that result by the particular effect of quinolones through trapping of DNA gyrase and topoisomerase IV on chromosomal DNA and/or by possible ROS attack [18,19], could not be released out of the bacterium since the cell wall would be intact initially, at least. In the case of gram-positive bacteria, it should be analyzed more confidently if DNA fragmentation is produced after β-lactam treatment, although more delayed than in gram-negative. If this is the case, despite of the effect, the thicker cell wall of gram-positive bacteria would also prevent the release of DNA fragments.

From the practical point of view, the background of DNA fragments was visualized without the necessity of incubation in lysing solution or any manipulation, so it could be used for a rapid determination of sensitivity or resistance, in liquid cultures. Nevertheless, the presence of the background could be indicative of susceptibility only in gram-negative bacteria, in those here assayed at least. Furthermore, the dilution of the culture modifies the density of the background, and different bacteria and different strains may show important differences in the amount of extracellular DNA fragments. A more confident discrimination between sensitive and resistant strains is achieved when also evaluating the cell wall response to the specific lysing solution.

The dose-response study shows that the β-lactam induces a progressive effect with increasing dose on the cell wall. This effect is evident even before the MIC dose, although it is very weak and seems not prevent growth of most of bacteria after removing the antibiotic. The cell wall damage is not homogeneous among cells, although a predominant level is observed for each dose. This level is more intense as dose increases. The heterogeneity in the effect on the cell wall is not mainly dependent on the growing stage since the cultures were exponentially growing when exposed to ampicillin. The background of DNA fragments appears to be observed at the MIC dose, and increases as dose increases, within the range of doses assayed.

The methodology has been confirmed as a rapid and simple procedure to distinguish susceptible and resistant strains of eight gram-negative and four gram-positive species, assaying four different β-lactams and vancomycin. The results were reproducible and accurate, in the 46 clinical strains. Although preliminary, the results are encouraging. Expanded work analysing many more strains is in progress. For example, links have been established between glycopeptide resistance and cell wall thickening in vancomycin-intermediate *Staphylococcus aureus *(VISA), as well as between macrolides and thickened cell walls in *S. aureus *[20,21]. These are interesting strains to be tested. Furthermore, the examination of the slides is going to be automated using a microscopy platform coupled with image capture and digital image analysis. This will allow a high-throughput examination of large numbers of microorganisms, for a rapid identification of resistant and susceptible strains to antibiotics that interfere with peptidoglycan biosynthesis.

## Conclusion

The technique to assess cell wall integrity may be a rapid and simple procedure to discriminate resistant and susceptible strains to antibiotics that interfere with peptidoglycan biosynthesis. The methodology may be useful not only at the clinical level but also to perform basic studies about the mechanisms of action of antibiotics that act at the cell wall.

## Methods

### Cultures, bacterial strains and experiments

In an initial approach to evaluate the procedure to determine cell wall integrity, ten clinical strains from *Escherichia coli*, isolated from urine samples in the microbiology service, were tested blind for susceptibility or resistance to amoxicillin/clavulanic acid. According to the Clinical and Laboratory Standards Institute (CLSI) criteria (susceptible: minimum inhibitory concentration - MIC ≤ 8/4; 8 μg/ml amoxicillin/4 μg/ml clavulanic acid; resistant: MIC ≥ 32/16; 32 μg/ml amoxicillin/16 μg/ml clavulanic acid), two strains were categorized as susceptible, five intermediate and three resistant. In this experiment, bacteria were growing in Mueller-Hinton agar at 37°C for 24 h. Then, they were diluted to an OD600 of 0.1 in Mueller-Hinton broth with 0, 8/4 and 32/16 μg/ml amoxicillin/clavulanic acid, incubated at 37°C for 60 min, and processed to determine cell wall integrity.

In a second experiment, the effect of the incubation time with the antibiotic was analyzed, after treatment with 8/4 and 32/16 μg/ml amoxicillin/clavulanic acid, in three clinical strains of *E. coli *isolated from urine samples, one susceptible (MIC: 8/4 μg/ml), one intermediate (MIC: 16/8 μg/ml) and one resistant (MIC: > 64/32 μg/ml). Moreover, it was tested both in cultures exponentially growing in Mueller-Hinton broth at 37°C, with aeration and shaking, and in cells cultured for 24 h in Mueller-Hinton agar dishes, as usual in the standard clinical microbiology laboratories. Cells were diluted to an OD600 of 0.1 in Mueller-Hinton broth, and incubated with the two doses of the antibiotic for 5, 10, 20, 30, 40, 60 and 75 min.

Thirdly, a dose-response experiment at the cell wall level of one *E. coli *strain isolated from an urine sample, susceptible to ampicillin (MIC: 4 μg/ml), was performed. Bacteria exponentially growing in Mueller-Hinton broth were diluted to an OD600 of 0.1 in Mueller-Hinton broth and then incubated for 60 min with 0, 1, 2, 4, 8, 12, 16 μg/ml ampicillin. Afterwards, the cultures were processed to determine viability and cell wall integrity. The halo size of the nucleoid was measured in 250-400 bacteria per dose after image capture and digital image analysis, and included in one of four qualitative categories: undamaged, with low cell wall damage, with high cell wall damage where the residual body of the bacterium was retained, and with high cell wall damage where the residual core from the bacterium was not recognized.

In a fourth approach, a total of 46 different clinical strains from eight different gram-negative and four gram-positive clinically relevant microorganisms, randomly selected in the clinical microbiology laboratory, were tested blind for susceptibility or resistance to one of four different β-lactams, either two penicillins (penicillin, ampicillin), or a third-generation cephalosporin (ceftazidime), or a carbapenem (imipenem), or vancomycin. Between 2009-2010 a total of 46 clinical isolates: *Enterobacteriaceae *(*Escherichia coli, Enterobacter cloacae, Klebsiella *spp.; including 2 *K. oxytoca*, *Morganella morganii, Proteus mirabilis, Salmonella *spp.), *Acinetobacter baumannii*, *Enterococcus *spp. (*E. faecalis *and *E. faecium*), and *Staphylococcus aureus *were collected from the A Coruña Hospital, NW Spain, and were included in the study (Table 1). Isolates were identified by API 20NE, API 20E, API 20STREP, and API STAPH (bioMérieux, Marcy l'Etoile, France) when appropriated. With *A. baumannii*, the identification was confirmed by molecular methods. Only one strain per patient was selected and in all cases bacterial isolates were associated with infection. All strains were isolated from urine samples (urinary tract infection), except those 7 from *A. baumannii*, 3 isolated from blood, 3 from respiratory samples, and 1 from wound infection. The microorganisms assayed, antibiotics employed and the CLSI breakpoint concentrations of susceptibility-resistance are presented in Table 1. Bacteria were grown for 24 h in Mueller-Hinton agar dishes. After dilution to an OD600 of 0.1, the bacteria were incubated with the CLSI breakpoint doses of susceptibility and resistance in Mueller-Hinton broth at 37°C, for 60 min and processed to determine cell wall integrity.

Cell growth in Mueller-Hinton broth was evaluated by monitoring turbidity at OD600 using a spectrophotometer (Unicam 8625, Cambridge, UK). The MIC was determined by automated microdilution (MicroScan Walkaway, Dade) or using the E-test (AB Biodisk, Solna, Sweden) according to manufacturer's instructions. Viability was determined by colony counting after sequential dilutions and plating.

### Determination of cell wall integrity

The Micromax^® ^kit (Halotech DNA SL, Madrid, Spain) had been designed to evaluate the integrity of the nucleoid from bacteria. Two new variants of the Micromax^® ^kit were used, one developed to assess the cell wall from gram-negative bacteria (Micromax^® ^WG-) and another one for gram-positive bacteria (Micromax^® ^WG+). An aliquot of each sample was diluted to a concentration of 5-10 million microorganisms/ml in Mueller-Hinton broth. The kit includes 0.5 ml snap cap microfuge tubes containing gelled aliquots of low-melting point agarose. The tube was placed in a water bath at 90-100°C for about 5 min to melt the agarose completely and then placed in a water bath at 37°C. Twenty-five microlitres of the diluted sample were added to the tube and mixed with the melted agarose. A 20 μl aliquot of the sample-agarose mixture was pipetted onto a precoated slide, and the sample was covered with a 22 × 22 mm coverslip. The slide was placed on a cold plate in the refrigerator (4°C) for 5 min to allow the agarose to produce a microgel with the trapped intact cells inside. The coverslip was removed gently, and the slide was immediately immersed horizontally in 10 ml of the lysing solution for 5 min at 37°C for gram-positive bacteria or at room temperature (22°C) in case of gram-negative bacteria. The slide was washed horizontally in a tray with abundant distilled water for 3 min, dehydrated by incubating horizontally in cold (-20°C) ethanol of increasing concentration (70%, 90%, and 100%) for 3 min each, and air-dried in an oven.

The dried slide was incubated in a microwave oven at 750 W for 4 min, and the DNA was stained with 25 μl of the fluorochrome SYBR Gold (Molecular Probes, Eugene, OR, USA) diluted 1:400 in TBE buffer (0.09 M Tris-borate, 0.002 M EDTA, pH 7.5) for 2 min in the dark, with a glass coverslip. After a brief wash in phosphate buffer pH 6.88 (Merck, Darmstadt, Germany), a 24 × 60 mm coverlisp was added and the slides visualized under fluorescence microscopy.

### In situ digestion with proteinase K and with DNase I

Many cultures sensitive to beta-lactams showed a diffuse microgranular-fibrilar background. To investigate the nature of this background, *in situ *digestion with enzymes and Fluorescence *In Situ *Hybridization (FISH) with a whole genome probe were performed.

One strain of *E. coli *susceptible to ampicillin, isolated from an urine sample, was incubated with this antibiotic (32 μg/ml) and another strain of *A. baumannii*, isolated from a respiratory sample, was incubated with imipenem (0.76 μg/ml), in Mueller-Hinton broth at 37°C for 60 min, with aeration and shaking. Afterwards, three microgels (18 × 18 mm) on each slide were prepared for each microorganism, as described before, but without the lysis step. One microgel corresponded to the control culture without antibiotic, and the other two, to the culture incubated with the antibiotic. Some slides were washed by immersion in proteinase K buffer (SDS 1%, EDTA 2 mM, pH 7.5) and some slides were washed in DNase I buffer (Tris-HCl 20 mM, MgCl_2 _2 mM, pH 8.3), three times, 5 min each. In the first case, whereas one of the microgels from the culture treated with the antibiotic was only incubated with the proteinase K buffer, the other microgel was incubated with proteinase K in buffer (2 mg/ml). In the case of the slides washed with the DNase I buffer, one of the microgels from the culture treated with the antibiotic was only incubated with the DNase I buffer and the other microgel was incubated with 2.5 U DNase I in buffer. Incubations were performed after covering with a glass coverslip, at 37°C, 30 min, in a humid chamber. Finally, the slides were washed in distilled water, dehydrated in increasing ethanol baths (70%-90%-100%) 5 min each, air dried and stained with SYBR Gold (1:400).

### Fluorescence In Situ Hybridization (FISH)

Fifty μl of each liquid culture of susceptible *E. coli *and *A. baumannii*, incubated with ampicillin and imipenem respectively as described previously, were mixed with 950 μl of methanol:acetic-acid (3:1), one drop being spread onto glass slides and air-dried. The slides were immersed in methanol:acetic-acid (3:1) 5 min and air-dried again. Then, they were incubated with increasing ethanol baths (70-90-100%), -20°C, 5 min each, and air-dried. DNA was denatured by immersion in 75% formamide/2 × SSC, pH7, 67°C, 90 sec and then the slides were immersed in increasing ethanol baths (70-90-100%), -20°C, 5 min each, and air-dried.

Whole genome DNA probes to label the total DNA from *E. coli *and from *A. baumannii *were prepared. DNA from each microorganism was isolated using standard procedures, and was labelled with biotin-16-dUTP, using a nick translation kit, according to the manufacturer's instructions (Roche Applied Science, San Cugat del Vallés, Spain). The DNA probes were mixed at 4.3 ng/μl in the hybridization buffer (50% formamide/2 × SSC, 10% dextran sulfate, 100 mM calcium phosphate, pH 7.0) (1 × SSC is 0.015 M NaCitrate, 0.15 M NaCl, pH 7.0). The probes in hybridization buffer were denatured by incubation at 80°C for 8 min and were then incubated on ice.

The DNA probe solutions (15 μl) were pipetted onto the denatured and dried slides, covered with a glass coverslip (22 × 22 mm) and incubated overnight at 37°C, in the dark, in a humid chamber. The coverslip was removed, and the slides were washed twice in 50% formamide/2 × SSC, pH 7.0, for 5 min, and twice in 2 × SSC pH 7.0, for 3 min, at 37°C. The slides were incubated with blocking solution (4 × SSC, 0.1% Triton X-100, 5%BSA) for 5 min, covered with a plastic coverslip, in a humid chamber, at 37°C. This solution was decanted, and the bound probe was detected by incubation with streptavidin-Cy3 (Sigma Chem, St Louis, MN, USA) in 4 × SSC, 0.1% Triton X-100, 1%BSA (1:200), covered with a plastic coverslip, in a humid chamber at 37°C. After washing in 4 × SSC, 0.1% Triton X-100, three times, 2 min each, slides were counterstained with DAPI (1 μg/ml) in Vectashield (Vector, Burlingame, CA).

### Fluorescence Microscopy and Digital Image Analysis

Images were viewed with an epifluorescence microscope (Nikon E800), with a 100× objective and appropriate fluorescence filters for FITC-SYBR Gold (excitation 465-495 nm, emission 515-555 nm), PI-Cy3 (excitation 540/25 nm, emission 605/55 nm) and DAPI (excitation 340-380 nm, emission 435-485 nm). In the experiment of dose-response to ampicillin, images were captured with a high-sensitivity CCD camera (KX32ME, Apogee Instruments, Roseville, CA). Groups of 16 bit digital images were obtained and stored as .tiff files. Image analysis used a macro in Visilog 5.1 software (Noesis, Gif sur Yvette, France). This allowed for thresholding, background subtraction, and measurement of the average halo width size of the nucleoids in μm, delimitated between the peripheral edge of the nucleoid and the external limit of the cell body. In the case of unrecognized cell body, the centroid of the nucleoid was considered as the internal reference point to measure the halo width of the spread nucleoid.

## Authors' contributions

RS and MT performed technical experiments and statistical analysis. JG participated in image acquisition and image analysis. GB participated in the design of the study and data analysis. MCF performed standard microbiological procedures. JLF conceived the study, participated in its design and coordination and wrote the initial draft of the manuscript. All authors read and approved the final manuscript.
